# Hypoxia Inactivates the VHL Tumor Suppressor through PIASy-Mediated SUMO Modification

**DOI:** 10.1371/journal.pone.0009720

**Published:** 2010-03-16

**Authors:** Qiliang Cai, Suhbash C. Verma, Pankaj Kumar, Michelle Ma, Erle S. Robertson

**Affiliations:** Abramson Comprehensive Cancer Center and Department of Microbiology, University of Pennsylvania School of Medicine, Philadelphia, Pennsylvania, United States of America; University of Hong Kong, Hong Kong

## Abstract

The hypoxic microenvironment contributes to embryonic development and tumor progression through stabilization of the potent transcriptional factor HIFα. In normoxia, the tumor suppressor protein VHL acts as an E3 ubiquitin ligase to target HIFα for proteolytic destruction. Increasing evidence shows that VHL is a multifunctional adaptor involved in inhibition of HIFα-dependent and independent cellular processes. However, the molecular effect of hypoxic stress on VHL functions remains elusive. Here we report that PIASy, a SUMO E3 ligase upregulated in hypoxia, interacts with VHL and induces VHL SUMOylation on lysine residue 171. Moreover, PIASy-mediated SUMO1 modification induces VHL oligomerization and abrogates its inhibitory function on tumor cell growth, migration and clonogenicity. Knockdown of PIASy by small interfering RNA leads to reduction of VHL oligomerization and increases HIF1α degradation. These findings reveal a unique molecular strategy for inactivation of VHL under hypoxic stress.

## Introduction

The ability of cells to recognize and respond to a low-oxygen environment (hypoxia) is critical in many physiological and pathological conditions [1], [2]. All mammalian cells express components of a conserved hypoxia response pathway [3]. The transcriptional factor HIF (hypoxia-inducible factor) is a central regulator of this pathway. HIF is a heterodimer that consists of an inducible α subunit and a constitutively expressed β subunit (also known as ARNT) [3]. To date, at least three HIFα (HIF1α, HIF2α, and HIF3α) have been identified that regulate transcriptional programs in response to low oxygen levels. Under normal oxygen tension, HIF1α is hydroxylated and rapidly targeted for proteasome-mediated degradation through the ECV (or VBC, namely elongin BC/Cul2/VHL) E3 ubiquitin ligase complex which requires the recognition of von Hippel Lindau (VHL) tumor suppressor [4]–[6]. When cells are exposed to a hypoxic environment, this hydroxylation-mediated degradation pathway is blocked, thereby allowing HIF1α to accumulate in the nucleus, where it binds to the constitutively expressed HIF1β and transactivates hypoxia-responsive genes that are implicated in cellular metabolism, angiogenesis, invasion, and metastasis [3], [7]. However, other studies suggest that VHL is also able to target HIF1α for destruction in an hydroxylation independent manner during hypoxia [8]. These studies indicated that VHL is a critical regulator of the ubiquitous oxygen-sensing pathway.

VHL was first identified as a tumor suppressor in 1993 [9]. Inactivation or loss of both VHL alleles has been widely demonstrated in the majority of sporadic clear cell renal carcinomas and cerebellar hemangioblastomas [10]. Reintroduction of wild type VHL into *VHL* ^−/−^ renal carcinoma cells (RCC) suppresses tumor formation *in vivo* [11]. In humans, the VHL gene encodes a 213-residue protein which contains two functional domains but neither with any known enzymatic activity [12]. The α domain binds to elongin C and the β domain acts as the substrate-docking interface for targeting proteins [13]. The best-characterized function of VHL is as an adaptor for targeting HIFα for proteolytic degradation [5], [6], and has been considered to be the major tumor suppressor activity associated with VHL. Nevertheless, although many of the HIFα-induced cellular responses undoubtedly contribute to tumor progression, alone they appear to be insufficient to induce tumor formation [12]. Notably, recent studies have demonstrated that VHL also negatively regulates several HIFα-independent transcriptional pathways, which includes targeting Rbp1 (the large subunit of the RNA polymerase II complex) for ubiquitylation in a similar manner to HIFα [14]; linking CKII kinase with CARD9 for inhibition of NF-κB pathway [15]; and stabilizing Jade-1 for proteasomal degradation of oncoprotein β-catenin [16]. These imply that VHL is a multipurpose adaptor protein that engages in multiple protein-protein interactions to control diverse HIFα-dependent and independent cellular processes. Thus, under hypoxic stress, inactivation or loss of VHL function appears to be an early and requisite step in tumor development. Exploring the regulatory mechanisms underlying VHL cellular functions in oxygen-deprived cells is likely to also be important.

The protein inhibitors of activated STAT (PIAS) proteins were originally reported as specific inhibitors of the STAT family of transcription factors [17], and more recently they have been identified as SUMO (a ubiquitin-like modifier) E3 ligases [18]. There are at least five mammalian PIAS proteins that have been identified so far, PIAS1, PIAS3, PIASx (α/β), and PIASy [18]. They all contain a conserved RING finger-like domain and have SUMO E3 ligase activity with differing target specificities [19], [20]. Similar to ubiquitin-like modification, PIAS-mediated SUMOylation is also reversible and dynamic and regulates diverse cellular processes, including protein targeting, stabilization, nuclear translocation, transcriptional control and stress response [18], [21], [22].

Here we identify PIASy as a specific negative regulator of VHL in response to hypoxic stress. We show that PIASy interacts with VHL and induces VHL SUMOylation on lysine 171, facilitating its oligomerization. This results in inactivation of the inhibitory function of VHL as a tumor suppressor via both HIF-dependent and independent ways.

## Results

### Lys-171 is the primary site for SUMO modification of VHL

**Figure 1:**
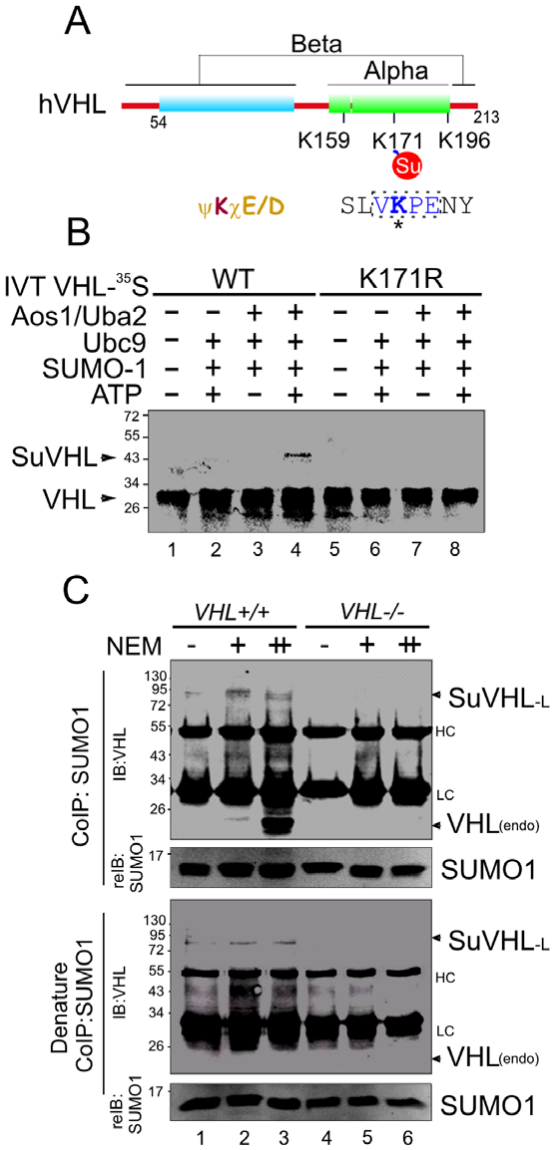
VHL can be SUMOylated *in vitro* and *in vivo*. (**A**) Schematic representation of VHL with three potentially modification Lysines K159, K171 and K196. K171 is the putative SUMOylation site based on SUMOplot™ analysis. Su, SUMO. (**B**) Wild type VHL but not its K171R mutant was SUMOylated by SUMO1 *in vitro*. *In vitro*-translated, radiolabeled VHL was incubated with the indicated recombinant proteins. Reaction mixtures were analyzed by SDS-PAGE and autoradiography. (**C**) SUMO1 modification of endogenous VHL. HEK293 and 786-O cells were individually treated with deSUMOylation inhibitor NEM (20 µM, 40 µM) for 1 hour before harvest. Cell lysates were subjected to native or denature immunoprecipitation with antibodies against SUMO1 (Y299), and immunoblot analysis with antibodies against VHL. The membranes were stripped and reblotted with SUMO1 antibodies. SuVHL, SUMOylated VHL; HC, heavy chain; LC, light chain.

Increasing evidence shows that SUMO1 modification is involved in regulation of HIF signaling pathway during hypoxia [8], [23], we wanted to determine whether VHL undergoes SUMO1 posttranslational modification. Analysis of the human VHL protein sequence indicated the presence of a VK_171_PE motif in the alpha domain that fits the conserved motif (ψKXD/E) for SUMO conjugation (Figure 1A) [24]. Furthermore, this motif and the adjacent residues are completely conserved in all VHL proteins from different species, including humans, monkey, dog, rat and mice (supplementary information, Figure S1). To determine whether lysine 171 residue is required for the SUMOylation of VHL, we performed an *in vitro* SUMOylation assay with wild type VHL and a mutant where the lysine at position 171 was replaced by arginine using site directed mutagenesis. ^35^S-labeled VHL proteins were incubated with Aos1/Uba2 (E1), Ubc9 (E2), SUMO1, and ATP which resulted in detectable SUMOylation of wt VHL but not of the K171R mutant (Figure 1B), suggesting that Lys-171 is the major SUMOylation site on VHL. To confirm that VHL is SUMO modified *in vivo*, we individually immunoprecipitated SUMO1 from native and denatured cell lysates of *VHL* positive or negative 786-O and analyzed the samples by immunoblotting with the specific antibody against VHL. Surprisingly, of the two major VHL-antibody-reacting bands (∼80 and 24 kDa; Figure 1C, top panel, lane 1 to 3) in VHL positive cells under native condition, and only the 80 kDa protein band (suVHL-L) but not a band with *in vitro* suVHL size was recognized by the VHL antibody under denaturing conditions (Figure 1C, bottom panel, lane 1 to 3). These data demonstrate that endogenous VHL with an apparent molecular weight of 80 kDa is conjugated with SUMO1, and that native VHL associates with SUMO1-modified VHL or other proteins with SUMO1 modification. The observation that SuVHL-L complex levels of native VHL are increased by treatment with the deSUMOylation inhibitor (NEM) further supports this conclusion, although it was not a dramatic increase when NEM was increased.

### PIASy interacts with VHL and induces VHL SUMOylation

**Figure 2:**
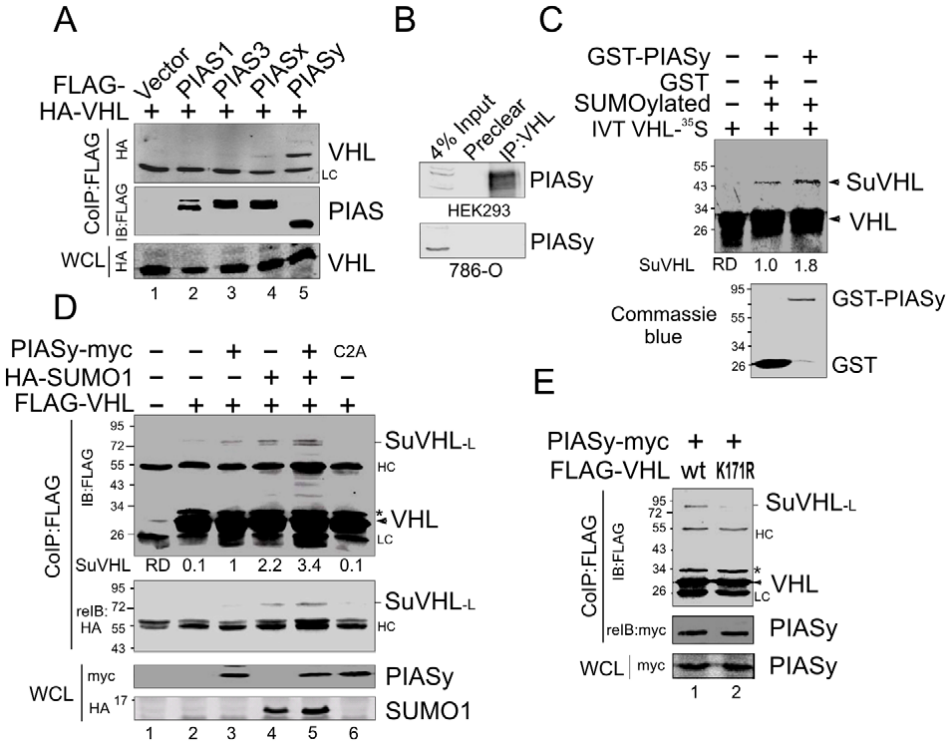
PIASy enhances SUMOylation of VHL on lysine 171. (**A**) PIASy interacts with VHL *in vivo*. HEK293 cells were cotransfected with expression plasmids as indicated in the figure. Forty-eight hours posttransfection, cell extracts were subjected to immunoprecipitated (IP) and immunoblotting (IB) as indicated. (**B**) Endogenous PIASy interacts with VHL. Preclear, normal rabbit serum. (**C**) PIASy enhances the conjugation of SUMO1 to VHL. *In vitro*-translated, radiolabeled VHL was subjected to SUMOylated assays as described in the Figure 1b in the presence of GST or GST-PIASy. Commassie staining shows the amounts of input GST recombinant protein. Relative intensity (RD) of SUMOylated VHL (SuVHL) is indicated. (**D**) RING domain of PIASy is required for SUMOylation of VHL. (**E**) PIASy-induced SUMOylation of VHL band (SuVHL) *in vivo* is abolished by lysine 171 mutation. HEK293 cells were individually cotransfected with expression vector encoding the indicated proteins in the top panel. Forty-eight hours posttransfection, cell extracts were subjected to immunoprecipitated (IP) and immunoblotting (IB) as indicated in the figure. WCL, whole cell lysate; SuVHL, SUMOylated VHL; HC, heavy chain; LC, light chain. The asterisk denotes uncharacterized protein band.

To explore the reason for the different size of VHL SUMOylation *in vitro* and *in vivo*, we next sought to identify whether a specific SUMO E3 ligase was involved in the process of VHL SUMOylation. Since the members of PIAS family are the major SUMO E3 ligases [18], we performed coimmunoprecipitation assays to determine whether VHL associates with one of the PIAS family members *in vivo*. Strikingly, we observed that only PIASy but not any other member (PIAS1, PIAS3, and PIASx) of the PIAS family strongly associated with VHL by coimmunoprecipitation assays (Figure 2A). The data from the immunoprecipitation assays targeting VHL, followed by immunoblotting for PIASy, showed that endogenous VHL indeed immunoprecipitated with PIASy (Figure 2B). To determine whether PIASy contributes to VHL SUMOylation, we performed *in vitro* SUMOylation assays in the presence or absence of GST fusion PIASy. The results showed that PIASy does increase the intensity of ∼43 kDa SUMO1-conjugation band of VHL *in vitro* (Figure 2C). Further experiments using coimmunoprecipitation assays showed that PIASy was able to enhance the affinity of SUMO conjugating enzyme Ubc9 bound to VHL (data not shown). Unexpectedly, in the coexpression system, the results of immunoprecipitation followed by immunoblotting showed that neither PIASy nor SUMO1 alone induces the ∼43 kDa band of SUMOylated VHL, but a migrating band which is similar in size to endogenous SUMOylated VHL (SuVHL-L) (Figure 2D, lane 3 and 4, compared with Figure 1C, lane 1 to 3). Moreover, in contrast to either PIASy or SUMO1 alone, the combination of both PIASy and SUMO1 further increased the intensity of SuVHL-L (Figure 2D, lane 5 compared with lane 3, 4). The site mutation of RING domain of PIASy resulted in the loss of PIASy activity on SuVHL-L (Figure 2D, lane 3 and 6), and thus indicated that the SUMO E3 ligase function of PIASy is required for PIASy-induced SUMOylation of VHL. Previous studies have reported that PIASy can interact with p53 [25], and that p53 can associate with VHL [26]. To exclude the possibility that p53 might be contributing to VHL SUMOylation, we also tested p53 deficient Saos-2 cells. Interestingly, PIASy does induce SuVHL-L in the Saos-2 cells and argues against p53 as a linker for PIASy and VHL interaction (data not shown). Furthermore, although PIASy bound equally to wild type VHL and its K171R mutant, PIASy only induced SuVHL-L band in wild type VHL instead of the K171R mutant (Figure 2E), indicating that SuVHL-L is indeed an isoform of VHL SUMOylated on Lys-171.

### PIASy-mediated SUMOylation of VHL facilitates oligomerization

**Figure 3:**
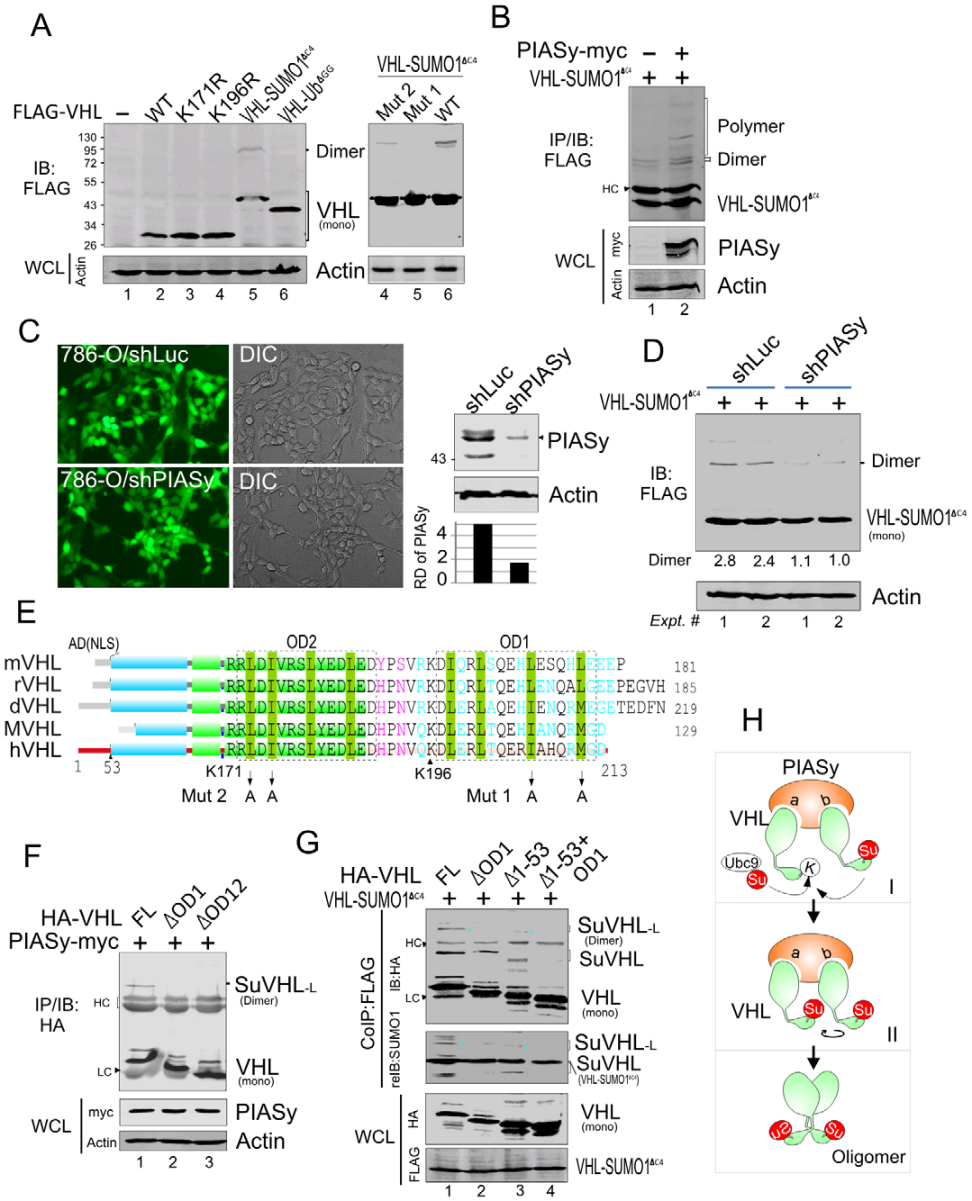
PIASy-mediated SUMOylation facilitates VHL oligomerization. (**A**) SUMO-1 modification of VHL increases VHL oligomerization *in vivo*. 786-O cells were cotransfected with expressing plasmids as indicated in the figure. At 48 hr posttransfection, cell extracts were subjected to immunoblotting with antibodies to FLAG and β-actin, respectively. (**B**) PIASy enhances oligomerization of VHL-SUMO1^ΔC4^ in 786-O cells. Cells were cotransfected with expressing plasmids as indicated in the figure, and performed oligomer assays by similar method as described in panel A. (**C**) Establishment of Lentivirus-mediated PIASy knockdown in 786-O cell lines. The GFP fluorescence shows the cells with PIASy (shPIASy) or luciferase (shLuc) knockdown after puromycin selection. The efficiency of PIASy knockdown was determined by immunoblotting with antibodies to PIASy, and the relative density (RD) of PIASy were showed in the right panel. Actin immunoblotting was used as the loading control. DIC, differential interference contrast. (**D**) Loss of PIASy represses oligomerization of SUMOylated VHL. 786-O cell lines with or without PIASy knockdown was individually transfected with VHL-SUMO1^ΔC4^ and performed immunoblotting for oligomerization assays. (**E**) The homology of two potential oligomerization domains (OD1 and OD2) of VHL from mice (mVHL), rat (rVHL), dog (dVHL), monkey (MVHL) and human (hVHL). The acidic domain (AD), two conserved functional domains (α and β) and high diversity carboxyl terminus is indicated. (**F**) The OD1 domain of VHL is required for PIASy-induced oligomerization. 786-O cells were cotransfected with expressing plasmids as indicated in the figure, and performed oligomer assay by similar method as described in panel A. (**G**) VHL-SUMO1^ΔC4^ associates with native VHL and induces OD1-dependent oligomerization of SUMOylated VHL. 786-O cells were cotransfected with expressing plasmids as indicated in the figure, and performed oligomer assay by similar method as described in panel A. The membrane of VHL-SUMO1^ΔC4^-immunoprecipitated complex followed by immunoblotting (IB) with HA antibody was stripped and reimmunbloted (reIB) with SUMO1 to present the SUMOylation position of native HA-VHL induced by VHL-SUMO1^ΔC4^. (**H**) A model of the role of PIASy functions on VHL oligomerization. PIASy potentially contains two VHL-binding sites and collaborates to induce VHL SUMOylation and oligomerization. HC, heavy chain; LC, light chain; WCL, whole cell lysate.

As the molecular mass of SuVHL-L induced by PIASy is about 2 times of mono-SUMOylated VHL, we hypothesized that SuVHL-L might be a dimer of mono-SUMOylated VHL, and that PIASy induces VHL SUMO1 modification resulting in its oligomerization. To directly address this point, we individually generated VHL-SUMO1^ΔC4^ by fusing the SUMO1 molecule to the carboxyl terminus of VHL with Lys171 mutation (as SUMO1 modification occurs within the carboxyl terminus of VHL, and to avoid other posttranslational modifications besides SUMOylation on Lys171), and the C-terminal glycines in charge of the covalent link were removed in SUMO portions to exclude the possibility of the fusion protein acting as a SUMO-like molecule. The VHL fusion with ubiquitin (VHL-Ub^ΔGG^) using the same strategy was used as a parallel control. We then detected the expression pattern of VHL -SUMO1^ΔC4^, VHL-Ub^ΔGG^, as well as the wild type VHL and its K171R or K196R mutants in 786-O cells. In agreement with our hypothesis, the immunoblotting results from the whole cell lysates showed that only VHL-SUMO1^ΔC4^ and neither wild type VHL nor its single lysine mutants (K171R or K196R) was able to undergo oligomerization as seen by the appearance of a dimer size band which is identical to the size of endogenous SuVHL-L (Figure 3A). Meanwhile, oligomerization of VHL-SUMO1^ΔC4^ was enhanced by PIASy coexpression (Figure 3B), and reduced by lentivirus-mediated loss of endogenous PIASy (Figure 3C, D), which highlights the role of PIASy as a critical player in oligomerization of SUMOylated VHL. To further prove that SUMO1 modified VHL can undergo oligomerization, we performed cross-linking assays by using the *in-vitro* translated proteins VHL, VHL-SUMO1^ΔC4^ and VHL-Ub^ΔGG^. Consistent with our findings, VHL-SUMO1^ΔC4^ showed increased oligomerization compared to wild type VHL or VHL-Ub^ΔGG^, and the extent of VHL-SUMO1^ΔC4^ oligomerization was similar to the oligomerization of the positive control p53 (supplementary information, Figure S2A). Unexpectedly, in the absence of the cross linker, the mixture of *in-vitro* translated VHL-SUMO1^ΔC4^ naturally produced the dimerized form, which had strong resistance to reduction by mercaptoethanol at high temperatures (supplementary information, Figure S2B), further supports our hypothesis that SUMO1 modification of VHL favors oligomerization.

### VHL oligomerization is dependent on Leucine-rich domain

In an effort to define the specific domain of VHL required for its oligomerization, we analyzed the amino acid sequence of VHL from different species, and noticed that there are two conserved Leucine-rich oligomerization domains (OD1 and OD2) linearly located at the carboxyl terminus of VHL (Figure 3E), similar to the domain of p53 required for oligomerization [27]. Using wild type VHL and its mutants with single (OD1) or double (OD12) deletion of oligomerization domains, we performed oligomerization assays in the presence of PIASy and found that deletion of the OD1 domain is sufficient to abolish oligomerization of VHL *in vivo* (Figure 3F). Furthermore, the intensity of SUMO1-modified VHL dimerization is greatly reduced when the Leucine residue is mutated in the OD1 domain compared to the OD2 domain (Figure3A, right panel). This indicates that SUMOylation-mediated oligomerization of VHL is dependent on its oligomerization domain. In addition, we also asked whether the SUMO-modified isoform of VHL will facilitate native VHL SUMOylation and oligomerization in an oligomerized domain (OD1)-dependent manner. We coexpressed HA tagged native or OD1 deleted VHL along with FLAG-tagged VHL-SUMO1^ΔC4^, and performed immunoprecipitation using FLAG antibodies. We found that FLAG-tagged VHL-SUMO1^ΔC4^ is not only efficiently associated with native HA-VHL, but also induced SUMOylation and dimerization of the HA-VHL isoform which is similar in size to the mono SuVHL and oligomerized SuVHL-_L_ protein, respectively (Figure 3G, lane 1). This evidence was further supported by the fact that the shorter isoform of HA-VHL with amino 53 residue deletion (Δ1-53) had a similar pattern (Figure 3G, lane 3). However, the two HA-VHL mutants (ΔOD1 and Δ1-53+OD1) lacking the oligomerization domain had little or no production of dimerized HA-VHL or other SUMO-modified isoforms, although VHL-SUMO1^ΔC4^ remained associated with VHL (Figure 3G, lane 2 and 4). This strengthens our hypothesis that SUMO modification of VHL facilitates native VHL SUMOylation and in turn oligomerization, and that PlASy plays a critical role on both VHL SUMOylation and oligomerization, and might contain two VHL-interacting sites in this process (Figure 3H).

### The RING domain is critical for PIASy induction of VHL oligomerization

**Figure 4:**
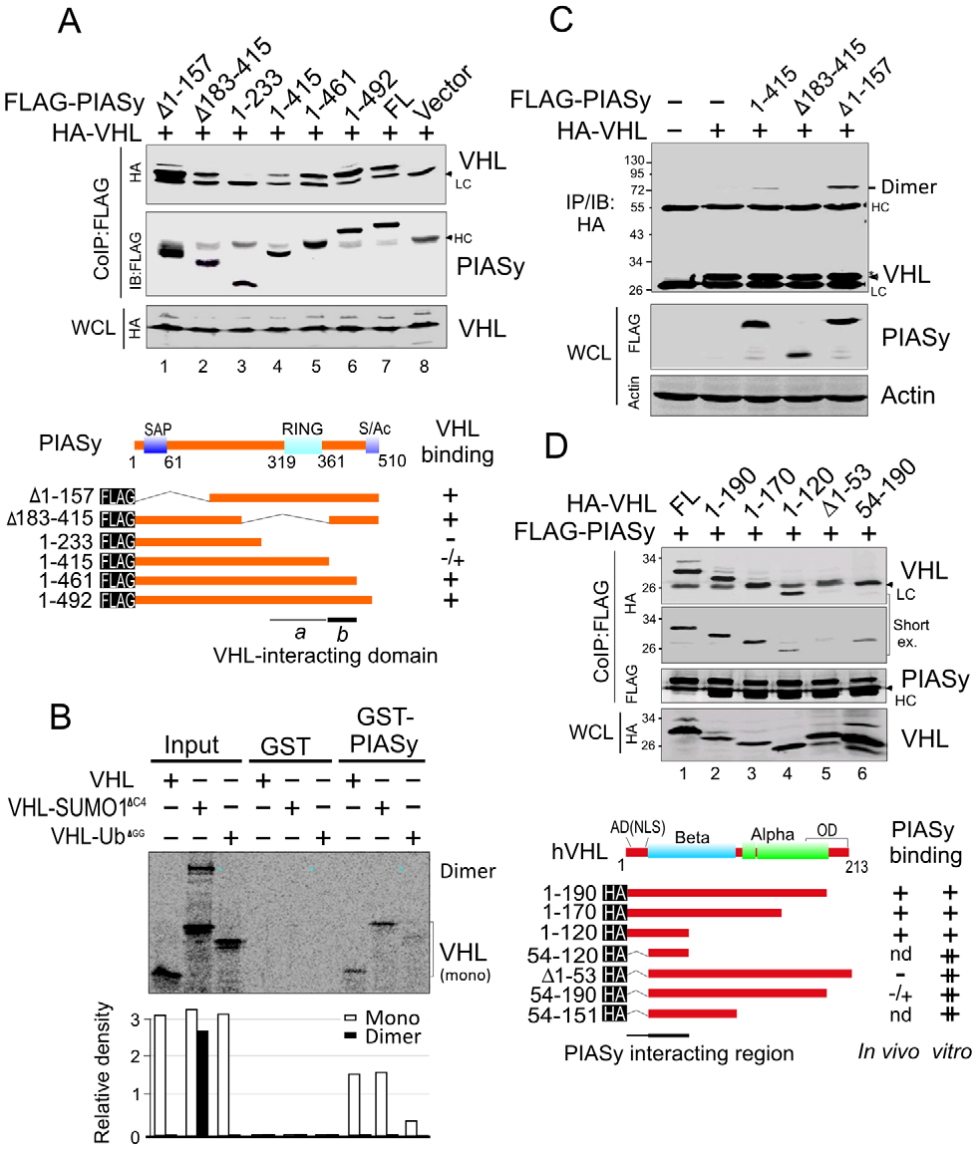
The functional domains required for PIASy and VHL interaction. (**A**) PIASy interacts with VHL through its carboxyl 46 amino acids. HEK293 cells were transfected with indicated expressing plasmids and harvested at 48 hr posttransfection. Cell extracts were subjected to immunoprecipitation (IP) and immunoblotting (IB) with antibodies as indicated. Schematic showing the binding affinity of PIASy truncated mutants with VHL (Bottom panel). (**B**) PIASy strongly interacts with native and SUMOylated VHL but slightly with dimerized or ubiquitylated VHL *in vitro*. *In vitro*-translated, radiolabeled VHL, VHL-SUMO1^ΔC4^ and VHL-Ub^ΔGG^ was individually incubated with GST or GST-PIASy recombinant proteins. Bound complexes were analyzed by autoradiography; Input, 10%. The relative density of binding affinity is shown in the bottom panel. (**C**) The carboxyl terminus including RING domain of PIASy is required to induce VHL oligomerization. 786-O cells were transfected with indicated expressing plasmids and then harvested at 48-hr posttransfection. Cell extracts were subjected to coimmunoprecipitated (CoIP) and immunoblotted (IB) as indicated. The asterisk denotes uncharacterized protein band. (**D**) Both acidic and beta domain are necessary for VHL to interact with PIASy in vivo. 786-O cells were transfected with indicated plasmids and then harvested at 48 hr posttransfection. Cell extracts were subjected to coimmunoprecipitated (CoIP) and immunoblotted (IB). The schematic showing the binding affinity of VHL truncated mutants with PIASy *in vivo* and *in vitro* (Bottom panel). HC, heavy chain; LC, light chain; WCL, whole cell lysate.

To determine which domain of PIASy is responsible for binding VHL, we generated six PIASy truncated mutants with or without the RING domain (Figure 4A). The mutant with the RING domain deletion (Δ183-415) maintained a strong affinity for VHL similar to that of full length PIASy (Figure 4A). Further deletions showed that the region 415 to 461 of PIASy was critical for association with VHL, although the central region (aa 233 to 415) showed similar binding affinity when compared to the complete loss of binding with the mutant containing only the first 233 residues (aa 1 to 233) (Figure 4A, lane 3, 4). This indicates that residues 415 to 461 are the critical amino acids important for interaction of PIASy with VHL. To further determine whether PIASy can directly bind to native, SUMO or ubiquitin modified VHL, we carried out *in vitro* pull-down assays. The results showed that both native and SUMO-modified VHL were able to bind directly to PIASy, whereas the ubiquitin-modified VHL showed much less affinity with PIASy (Figure 4B). Intriguingly, when compared to the monomer, the dimerized SUMO-modified VHL showed a significantly less capability of binding to PIASy (Figure 4B). This suggests that PIASy binds with strong affinity to native and SUMO-modified VHL but had little or less affinity for the ubiquitin-modified or dimerized SUMO-modified VHL (supporting the proposed model in Figure 3H).

To determine whether the VHL-interacting domains of PIASy are required for stimulating VHL oligomerization, we performed oligomerization assays by using HA-tagged VHL in the presence of different PIASy truncated mutants which includes either VHL-binding domain *a* (aa 183 to 414, RING domain), *b* (aa 415 to 461) alone or both. Strikingly, the results showed that neither VHL-binding domain *a* nor *b* alone, but the intact PIASy of both *a* and *b* was capable of inducing VHL oligomerization (Figure 4C). Therefore, it is noteworthy that the carboxyl terminus which includes both VHL-interacting domains *a* and *b* were required for PIASy induction of VHL oligomerization.

### PIASy binds to the β domain of VHL

Because VHL is able to directly interact with PIASy *in vitro*, we also asked which domain of VHL is required for association with PIASy. Based on the conserved and functional domains of VHL seen in different species [13], we generated a series of VHL truncated mutants fused with an HA tag (Figure 4D). Using *in vitro* translated VHL proteins, we performed the GST-PIASy *in vitro* pull down assays. Surprisingly, the data showed that the minimal truncated mutant of VHL β domain (aa 54 to 120) maintained significant binding activity with PIASy when compared with the negative control luciferase (supplementary information, Figure S3). Moreover, all mutants deleted for the acidic domain showed higher binding affinity to PIASy when compared to the full length VHL (supplementary information, Figure S3, and Figure 4B). This suggests that the acidic domain may affect the physical binding of VHL with PIASy *in vivo*. To determine the role of the acidic domain in the context of the association of VHL with PIASy, we performed coimmunoprecipitation followed by immunoblotting assays by using HEK293 cells coexpressing FLAG-PIASy and the individual VHL truncated mutants. The results showed that although the minimal truncated mutant of VHL (54-120) can physically interact with PIASy *in vitro*, there was no significant protein of the mutant with acidic domain deletion (Δ1-53) precipitated by PIASy *in vivo* (Figure 4D). This indicated that in addition to residues 54 to 120, the acidic domain is also required for interaction of PIASy with VHL *in vivo* (Figure 4D, bottom panel).

### PIASy is upregulated by hypoxia stress to block VHL-mediated degradation of HIF1α

**Figure 5:**
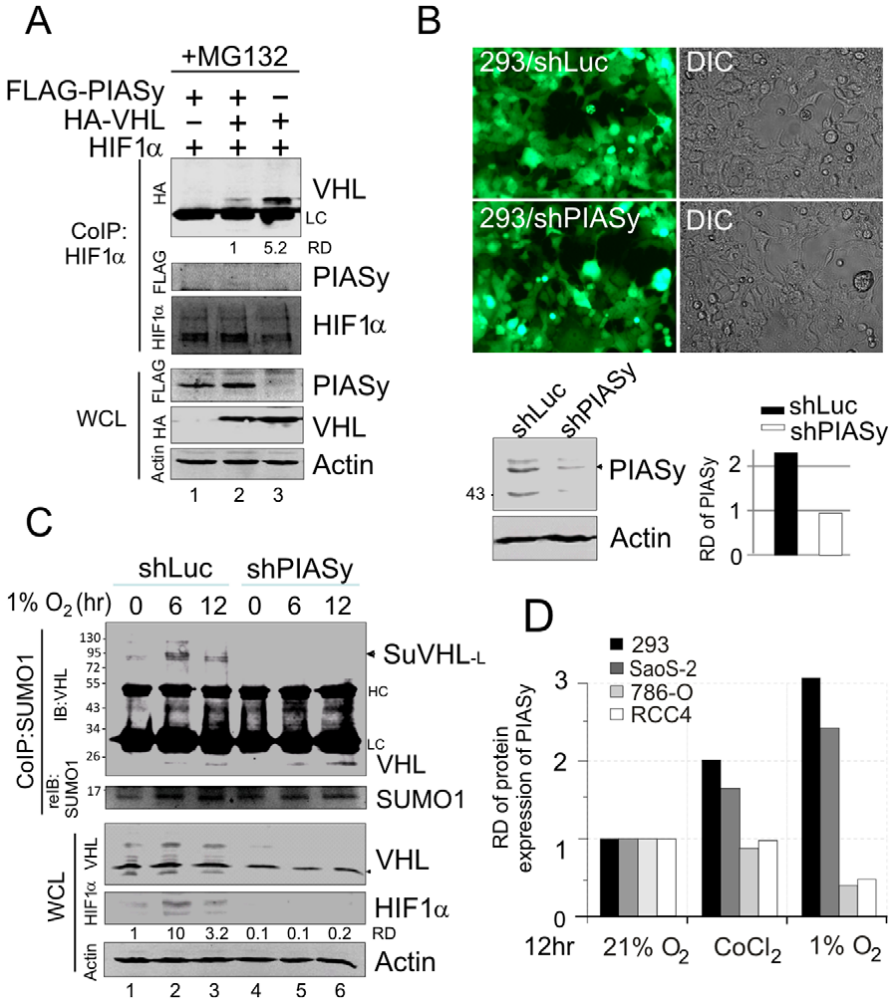
PIASy expression is upregulated by hypoxia for disruption of VHL-mediated HIF1α degradation. (**A**) PIASy competes with HIF1α to bind VHL. 786-O cells were transfected with expressing plasmids as indicated. Forty eight-hours posttransfection, cells were treated with 20 µM MG132 for 2 hr before harvested. Cell extracts were subjected to coimmunoprecipitated (CoIP) with anti-HIF1α and immunoblotted (IB) as indicated in the figure. Bound proteins were detected by anti-HA and anti-HIF1α immunoblotting. The membrane was stripped and reimmunoblotted (reIB) for anti-FLAG. Relative intensity (RD) of VHL bound to HIF1α is quantitated. (**B**) Establishment of Lentivirus-mediated PIASy knockdown in 293 cell lines. The GFP fluorescence shows the cells with PIASy (shPIASy) or luciferase (shLuc) knockdown after puromycin selection. The efficiency of PIASy knockdown was determined by immunoblotting with antibodies to PIASy, and the relative density (RD) of PIASy were showed in the right panel. Actin immunoblotting was used as the loading control. DIC, differential interference contrast. (**C**) Loss of PIASy reduces SUMOylation of VHL and in turn inhibits hypoxia-induced HIF1α. 293 cells with PIASy stable knockdown or control were cultured in 1% O_2_ for 0, 6, and 12 hours. Cell lysate were subjected to coimmunoprecipitation against with VHL followed by immunoblotting with SUMO1, or directly performed immunoblotting with antibodies to VHL, HIF1α and β-actin. (**D**) Protein level of endogenous PIASy is upregulated in hypoxia. Cells were treated with or without hypoxia (1% O_2_ or CoCl_2_) for 12 hours. Whole cell lysates were subjected to immunoblotting with anti-PIASy. The relative density (RD) was calculated by the quantitation of native band of PIASy in hypoxia compared to in normoxia. Intensity of Actin was used as internal control to normalize.

Because PIASy binds to the β domain of VHL which might overlap with the docking site for HIFα, we wanted to determine whether PIASy impairs the ability of VHL bound to HIFα by competing for binding to VHL. We coexpressed HIF1α with VHL in the presence of PIASy in 786-O cells, and monitored the levels of VHL in the HIF1α immunoprecipitated complex when treated with the proteasomal inhibitor. The results showed that the ability of VHL to bind with HIF1α was dramatically reduced in the presence of PIASy (Figure 5A, compare lane 2 with 3). Critically, PIASy did not associate with HIF1α (Figure 5A, lane 1).

To address whether the molecular effect of PIASy on VHL is required for HIFα stabilization in hypoxia, we determined the endogenous levels of both SUMOylated VHL and HIFα in 293 cells with or without PIASy repression at different time points under low oxygen conditions. As expected, in the PIASy-repressed cells, SUMOylated VHL (SuVHL-_L_) was significantly reduced along with a simultaneous loss of HIFα accumulation during hypoxia (Figure 5B, C), which is supported by the evidence that loss of PIASy results in less stability of SUMO1-modified VHL (VHL-SUMO1^ΔC4^) under prolonged hypoxia (supplementary information, Figure S4A). In contrast to the parallel treated group without PIASy repression, hypoxia-induced HIF1α was clearly detected and SUMOylated VHL (SuVHL-_L_) showed a dramatic increase in hypoxia compared to normoxia, although prolonged hypoxia exposure (>6 hr) did not further increase the intensity of SUMOylated VHL (Figure 5B, C). This suggests that PIASy-induced SUMO modification of VHL is stable within short term (<6 hr) rather than long term (>12 hr) hypoxia exposure. Consistent with the hypothesis that PIASy stabilizes HIFα, the results from the luciferase reporter assays with a reporter construct containing three copies of wild type or mutated HIFα-binding element (wHRE, mHRE) along with small interfering RNA against PIASy or Ubc9, showed that loss of PIASy leads to a specific reduction in the transcriptional activity of wild type HIFα-responsive element (supplementary information, Figure S4B). Furthermore, we also found that the protein expression of endogenous PIASy was upregulated about 2 to 3 fold by both low oxygen (1%) and chemical (CoCl_2_)-mimetic hypoxia in VHL-positive cell lines, but showed little or no change in VHL-negative cell lines (Figure 5D). These results suggest that PIASy is a critical molecule which contributes to regulation of VHL in response to hypoxic stress.

### PIASy-mediated SUMO modification of VHL abolishes its inhibitory functions on HIF1α

**Figure 6:**
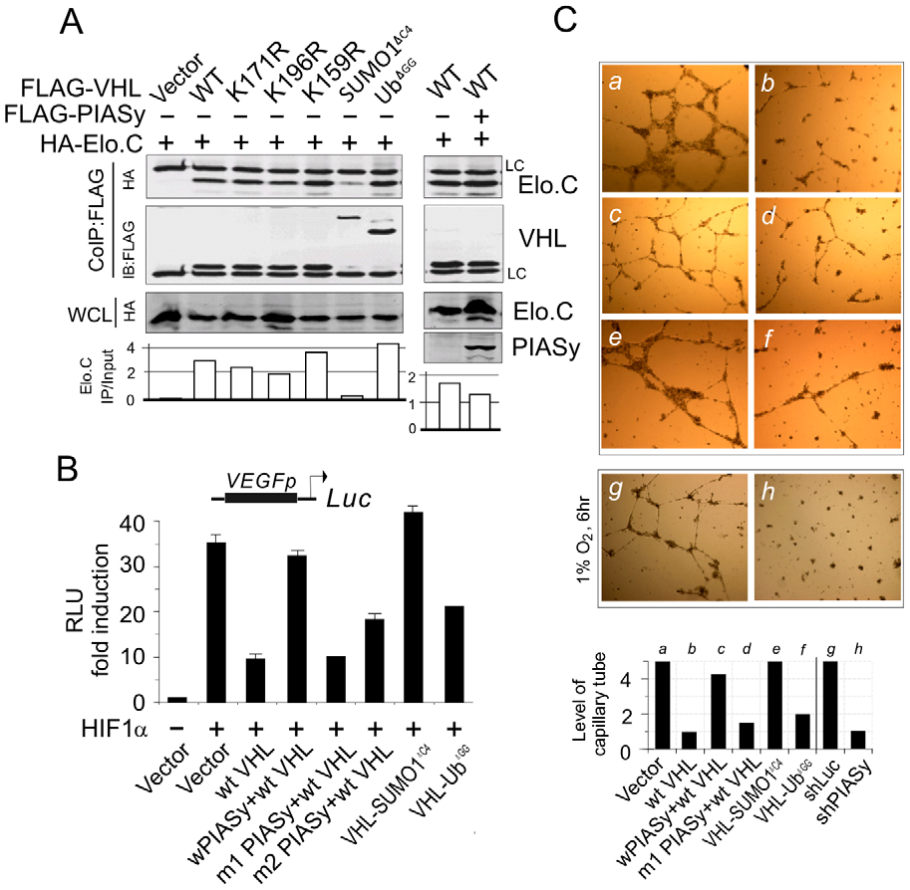
The SUMO1-modified isoform of VHL inactivates its inhibitory ability on the transcriptional activity of HIF2α. (**A**) SUMO1-modified isoform of VHL reduces its ability to associate with Elongin C. 786-O cells were transfected with expressing plasmids as indicated. Forty-eight hours posttransfection, whole cell extracts were subjected to coimmunoprecipitated (CoIP) and immunoblotted (IB) as indicated in the figure. (**B**) The effect of PIASy-mediated SUMO modification of VHL on the transcriptional level of VEGF promoter-luciferase reporter. 786-O cells were cotransfected VEGFp-luciferase reporter (5 µg) with VHL expressing plasmids (10 µg) in the presence or absence of PIASy (w, wild type; m1, mutant Δ183-414; m2, mutant C2A) as indicated. Total equal DNA amounts were made up by empty vector. The equal each protein expressions was confirmed by immunoblotting. Data are presented as means±SD of three independent experiments. (**C**) PIASy- mediated SUMOylation of VHL reverses the inhibitory effect of VHL on endothelial tube formation *in vitro*. The conditioned medium from 786-O cells expressing vector, wt VHL, wt VHL and PIASy (m1, PIASy mutant Δ183-414,), VHL-SUMO1^ΔC4^, or VHL-Ub^ΔGG^, or from PIASy-knockdown (shPIASy) or luciferase (shLuc) control 293 cells with 6 hr low oxygen (1% O_2_) treatment, was tested for tube formation assay *in vitro*. Photographs were taken at 24 hr post-incubation. The quantitation presents the average of the pattern/value association criterion from 5 random view fields per well.

To further elucidate whether PIASy-induced SUMO1 modification of VHL results in impaired formation of VHL ubiquitin complex, we performed coimmunoprecipitation assays to determine the binding affinity of Elongin C (the key component of VHL ubiquitin complex for targeting HIFα to degradation) with wild type VHL and its mutants (including VHL-SUMO1^ΔC4^ and VHL-Ub^ΔGG^) in the presence or absence of PIASy in *VHL*-deficient 786-O cells. The results showed that SUMO1 and not ubiquitin modification of VHL dramatically attenuates the ability of VHL to bind to Elongin C (Figure 6A, left panel), whereas, there was little or no effect on VHL with the single lysine mutation or when coexpressed with PIASy (Figure 6A). This indicates that posttranslational SUMO1 modification blocks the ubiquitin E3 ligase function of VHL on HIFα degradation via suppression of the VHL interaction with Elongin C.

To further corroborate this point, we determined the effect of wild type VHL in the presence or absence of PIASy, as well as its modified isoform (VHL-SUMO1^ΔC4^ and VHL-Ub^ΔGG^) on the transcriptional activity of HIF1α in 786-O cells by co-introducing VEGF (a key downstream molecule regulated by HIF) promoter-driven luciferase reporter. The results of reporter assays showed that wild type PIASy but not their RING domain mutant (m1 and m2) dramatically reverses the inhibition of VHL on the transcriptional activity of HIF1α on VEGF promoter (Figure 6B). Moreover, the transcriptional activity of HIF1α in SUMO1-modified VHL (VHL-SUMO1^ΔC4^) group had greatly increased as compared with wild type VHL, although the Ubiquitin-modified isoform (VHL-Ub^ΔGG^) also to some extent reverses inhibition of VHL on HIF1α (Figure 6B). This indicates that PIASy-mediated SUMOylation of VHL abrogates its inhibitory functions on HIF1α. Concordantly, *in vitro* endothelial tube formation assay using conditioned medium from the supernatants of the VHL or VHL/PIASy -expressing 786-O cells, showed that both PIASy coexpression and SUMO1 modified VHL markedly reduced the inhibitory ability of wild type VHL on tubule formation (Figure 6C, panels c and e), instead of the RING domain mutant of PIASy or Ubiquitin modified isoform (Figure 6C, panels d and f). Meanwhile, the results of condition medium from the supernatants of PIASy-repressed cells with 6 hr hypoxia treatment showed less ability to induce tube formation (Figure 6C, middle and bottom panels), further suggests that PIASy-mediated SUMOylation of VHL indeed inactivates native VHL and rescued HIFα from degradation.

### The SUMO1 modification of VHL inactivates its other HIF1α-independent tumor suppressor functions

**Figure 7:**
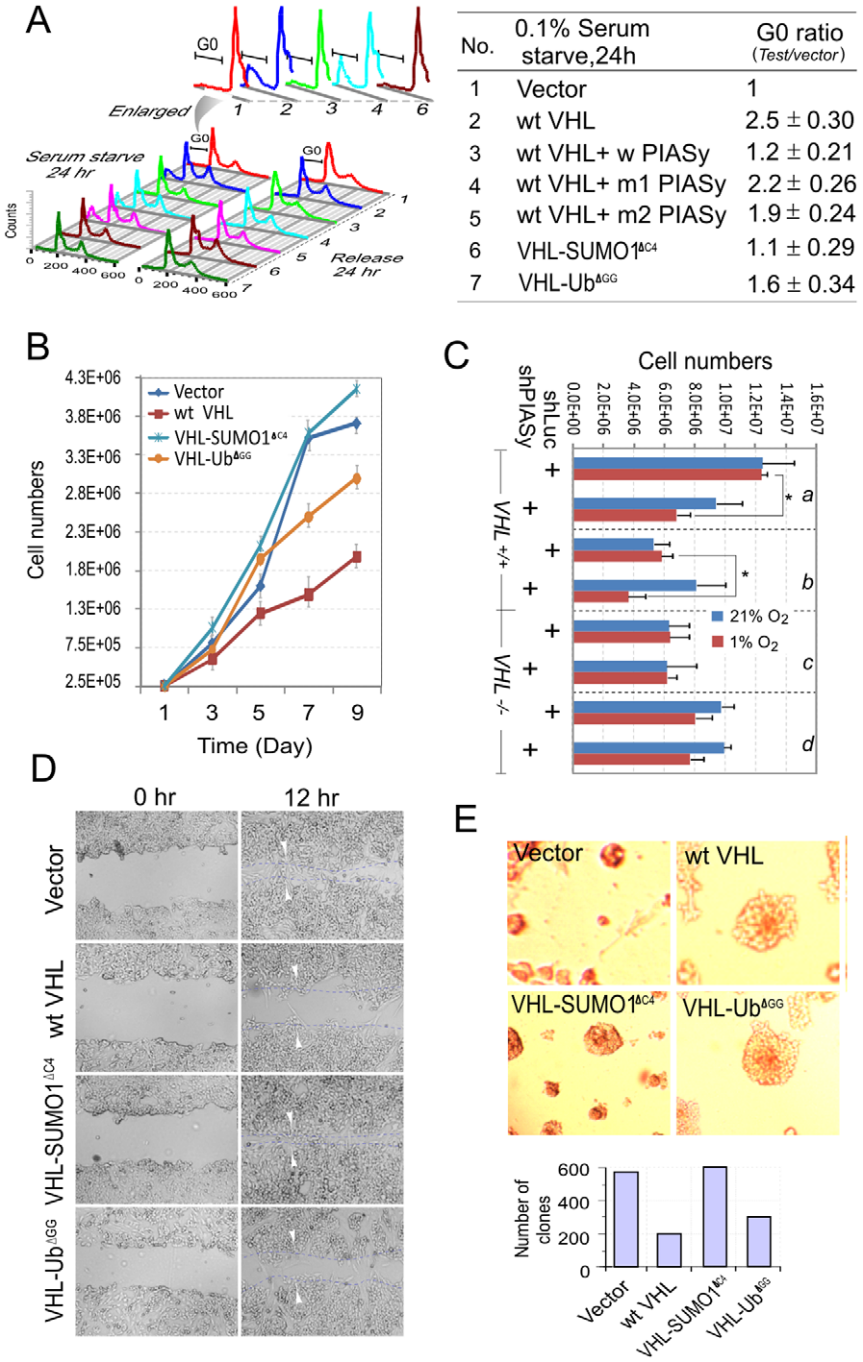
The SUMO1-modified isoform of VHL blocks its tumor suppressor function. (**A**) SUMO1-modified isoform of VHL eliminates the regulation of VHL on cell cycle under serum starve condition. 786-O cells were transfected with VHL expressing plasmids in the presence or absence of PIASy (w, wild type; m1, mutant Δ183-414; m2, mutant C2A) as indicated. At 24 hr posttransfection, cells were plated and incubated for 24 hrs in 0.1% serum, followed by incubation in 10% serum for 24 hrs or directly analyzed by flow cytometry after staining with propidium iodide. The relative ratios of G0 phase in each group under serum starve condition were showed by number compared to vector only. The data presents from three independent experiments. (**B**) Growth curves of 786-O cells expressing wild type VHL or its modified isoforms. The values are shown as means from two independent experiments. (**C**) Effect of hypoxia on the growth of *VHL* positive and *VHL* negative expressing cell lines with or without PIASy stable knockdown. The equal amount of each cell line was seeded. The cell numbers were counted at two days with either 21% or 1% oxygen treatment for 18 hours. *a*, HeLa; *b*, 293; *c*, 786-O; *d*, RCC4. (n = 3 assays/group, *p<0.05). (**D**) The cell mobility of wild type VHL and its modified isoform was determined by wound migration assay. The VHL-SUMO1^ΔC4^-expressing cells spreading along the wound edges are significantly slower, compared with the wild type VHL-expressing cells. All experiments were performed in triplicate. (**E**) SUMO1 but not ubiquitin modification of VHL affects the growth of RCC cells in a 3D growth assay. Digital images (×40) of the indicated and representative 786-O subclones grown as spheroids in semisolid media were captured on day 3. After ten days, colonies were counted and presented in the bottom.

To ascertain if SUMO modification is sufficient to inactivate the inhibitory functions of VHL on tumor cells, we tested whether PIASy-induced SUMO1 modification of VHL resulted in dysregulation of VHL as the cells exit the cell cycle on serum deprivation. Similarly, 786-O cells were transfected with vector, vector expressing wild-type VHL in the presence or absence of PIASy (wild type or mutants), or its modified isoform VHL-SUMO1^ΔC4^ and VHL-Ub^ΔGG^. In 10% serum, no difference in DNA content was observed between vector and wild type VHL or its modified isoforms, showing the similar growth characteristics for all cell lines (data not shown). However, when cells were switched to 0.1% sera for 24 hr, the DNA content of 786-O (Vector) cells was not significantly changed (Figure 7A, lane 1), whereas a dramatic increase in G0 DNA content was observed with the 786-O(wt) cells (Figure 7A, lane 2). Meanwhile, when coexpression with wt PIASy but not its RING domain mutants, VHL showed a great decrease in G0 DNA content, and the SUMO-modified isoform of VHL showed the most efficient inhibition of G0 arrest induced by wt VHL, which was almost the same as vector control (Figure 7A, lane 6 and 3 compared with lane 1). By contrast, readdition of 10% serum did not significantly alter the DNA content of the 786-O (vector, wt, or mutant) cells, suggesting that PIASy-mediated SUMO modification of VHL effectively blocked the inhibitory function of VHL on cell cycle progression in low serum. Furthermore, analysis of endogenous VHL levels, gene silencing of PIASy also specifically increased G0 arrest in the *VHL* positive cells with low serum treatment, but not in *VHL* negative cells (supplementary information, Figure S5A), suggesting that SUMO modification not only inactivated the control of VHL on cell cycle arrest but also increased cell cycle progression in hypoxia. To support this data, cell proliferation assays were performed using stably transfected wild type VHL and its mutants in 786-O cells. The results further showed that 786-O (VHL-SUMO1^ΔC4^) cells grew faster than 786-O (vector) cells, whereas 786-O (wt) cells were dramatically slowed (Figure 7B). Unlike in normoxia, gene silencing of PIASy consistently showed a decrease in proliferation of *VHL* positive cells but not *VHL* negative cells in hypoxia (Figure 7C), further corroborating the above data that PIASy-mediated SUMOylation of VHL does affect cell cycle progression during hypoxia.

To determine whether the inhibitory function of VHL on cell migration is also impaired by SUMO modification, we performed scratch wound assay to assess the effect of SUMO modification on the inhibition of VHL on 786-O cell motility. The results showed that the wild type VHL-expressing cells spread along the wound edges significantly slower than vector-transfected cells. However, the VHL-SUMO1^ΔC4^-expressing cells had similar motility compared to the vector-transfected cells (Figure 7D). Concordantly, loss of PIASy suppresses the cell migration of VHL positive cells (supplementary information, Figure S5B), indicating that SUMO modification abolished the inhibitory effect of VHL on tumor cell migration. In addition, as it is known that 786-O cells devoid of VHL grow as very compact and cohesive spheroids, while VHL-reintroduced 786-O cells grow as loosely arranged spheroids [28], [29], we compared the 786-O cells with wild type VHL or its mutants. The results showed that among the wt VHL and its mutants expressing 786-O cells, multiple SUMO-modified VHL (VHL-SUMO^ΔC4^)–expressing 786-O subclones grew as compact spheroids and closely resembled 786-O cells devoid of functional wt VHL (Figure 7E). Thus, SUMO modification of VHL is necessary for tumor cell growth in three-dimensional space mimicking the *in vivo* multicellular tumor growth conditions.

## Discussion

**Figure 8:**
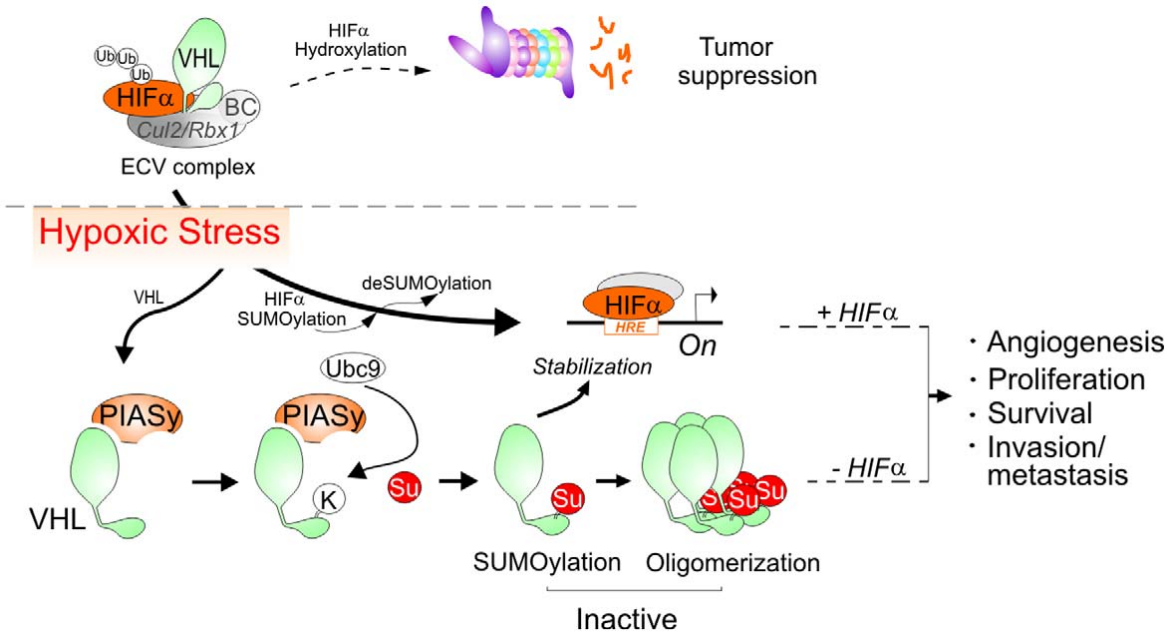
A model for the role of PIASy on VHL in response to hypoxic stress. Hypoxia enhances the interaction of PIASy with VHL, and induces PIASy-mediated VHL SUMOylation and oligomerization, and in turn results in the inactivation of VHL regulation in HIFα-dependent and independent pathways, which include angiogenesis, cell survival and proliferation, and cell mobility. However, loss of PIASy increases the VHL-mediated ECV (Elongin BC-Cullin 2/VHL) E3 ubiquitin complex targeting HIFα for ubiquitylation and proteasome-mediated degradation in the presence or absence of oxygen, and eventually leads to tumor suppression.

Although the ability of VHL to target HIFα for proteolysis as a ubiquitin E3 ligase is one of its major function [5], increasing evidence have demonstrated that VHL is a multipurpose adaptor for involvement in the inhibition of angiogenesis, cell cycle exit and fibronectin matrix assembly in a HIF-dependent or independent manner [30]–[32]. The overexpression of HIFα alone is insufficient to induce tumor formation in some cases [12], and genetic mutation of VHL leads to a range of human diseases [33]. Thus, it is highly possible that hypoxia may also inactivate VHL function as a tumor suppressor besides stabilizing HIFα by blocking hydroxylation. Although a great deal of effort has been put into studying the molecular mechanism of hypoxia stress on HIFα stabilization via posttranslational regulation [8], [23], [34]–[36], it still remains unclear whether hypoxia affects overall VHL tumor suppressor function. Recently, some studies have shown that in addition to genetic mutation the function of ubiquitin ligases (like VHL) can be dysregulated by controlling the ligase activity or the substrates using a number of strategies including posttranslational modifications, interactions with regulatory factors, or subcellular localization [37]. In this report, we now showed that PIASy, a SUMO E3 ligase, is up-regulated by hypoxia and stimulates VHL SUMOylation on lysine residue 171, which facilitates VHL oligomerization and inactivates VHL as a tumor suppressor in both a HIFα-dependent and independent strategy (Figure 8). Despite increasing reports showing that hypoxia induces HIF1α SUMOylation, and HIF1α stabilization depends on the deSUMOylation enzyme SENP1 [8], [38], we now provide an additional mechanism which shows that hypoxia also upregulates PIASy expression and inactivates VHL by promoting PIASy-mediated SUMOylation and oligomerization. This disrupts VHL ability to target HIF1α for degradation, and suggests that the oxygen-dependent recognition and binding of HIF1α by VHL might be precisely timed in that PIASy affects the engagement of VHL to either ECV E3 ubiquitin complex or other protein complex, thereby establishing a critical role for PIASy to promote the SUMOylation and oligomerization of VHL in a temporally coordinated manner, thus leading to inactivation of VHL function as a tumor suppressor in a HIFα-dependent and independent manner during hypoxia.

To date protein SUMOylation has emerged as an important strategy for modification of many proteins that play regulatory roles in diverse cellular processes, including protein relocalization, stability and stress response [22]. The PIAS family of SUMO E3 ligases has two domains that are crucial for SUMOylation: the RING finger domain that binds to Ubc9 and the SUMO-interacting motif that binds to SUMOs [22]. The RING finger domains of the PIAS proteins are indispensable for their SUMO E3 ligase activity [22]. In our study, although the deletion mutant of either RING domain or SUMO-interacting motif of PIASy (Δ183-415 and 1-461) retains the ability to associate with VHL, both of these two domains are required for PIASy-mediated *in vivo* VHL SUMOylation and oligomerization (Figure 4A and C), further supporting the notion that both RING finger and SUMO-interacting domains are critical for targeting proteins to be SUMO modified. More importantly, these results demonstrated that PIASy is unique in its mode of action when compared to other PIAS members in terms of its ability to interact with VHL and respond to hypoxia. Our data add to the growing evidence showing that posttranslational modifications can play a major role in regulating the activity of VHL, and raise the possibility that PIASy is a key regulator which responds to different cellular environments, including genotoxic and hypoxic stress [39], through the targeting of specific molecules (like NEMO and VHL), respectively.

Our data suggested that VHL undergoes oligomerization in response to hypoxic stress. However, this is not a unique example for cells in response to environment stress. In the oxidative environment, it has been found that the membrane protein MG53 undergoes oligomerization to initiate the assembly of the membrane repair machinery [40]. Another well-defined example is that oligomerization of p53 in the MDM2 complex in association with other molecules (like ATM and BAK) in response to DNA damage [41], [42]. Importantly, distinct from the way of other molecules forming oligomers, we found that VHL oligomerization is a result of its SUMO1 rather than its ubiquitin modification. Moreover, the carboxyl terminus of VHL contains an oligomerized domain which is similar to the oligomerized sequence of p53 [43], [44], although the native VHL forms much less oligomers than native p53 by *in vitro* cross-linking (supplementary information, Figure S2A). The evidence of high-level oligomerization of the SUMO1-modified VHL (VHL-SUMO^ΔC4^) in the *in vitro* cross-linking assay, suggests that VHL has a unique space structure and requires SUMO modification to alter its conformation before oligomerization. In addition, as shown in Figure 3E, the highly conserved sequence of the oligomerization domain among VHL homologs from different species suggests that the process of VHL oligomerization may be important for reversible regulation of VHL function. To our knowledge, this strategy by which hypoxic stress affects posttranslational modification of VHL provides a potentially novel alternative molecular mechanism for altering the function of critical cellular regulator under environmental stress.

Lysine residues act as acceptors not only for SUMO modification but also for all other Ubls (methylation and acetylation) [22]. It is therefore possible that SUMO conjugation could block other lysine dependent modification such as ubiquitylation. Previous studies have shown that VHL can be Neddylated on lysine 159 to associate with fibronectin matrix assembly and suppression of tumor development [28], [45], however, this posttranslational modification is not related to its E3 ligase activity. In our study, we have demonstrated that VHL loses its ability for HIFα modulation through SUMOylation and oligomerization through the action of the SUMO E3 ligase PIASy. The consequences of this modification include: 1) Conjugation of VHL to SUMO1 which alters protein-protein interaction with its substrates; and 2) Competition with other posttranslational modifiers. In support of this, we also demonstrated that VHL is also ubiquitylated on Lysine 171 in addition to SUMOylation (data not shown). Thus, SUMO1 modification can compete with VHL ubiquitylation for the major conjugation site at Lys171. It was previously shown that similar competition between SUMOylation and ubiquitylation occurs at the Lys-21 of IκBα [46]. This active interplay between SUMOylation and ubiquitylation is likely to dictate the degree of VHL stability, and SUMO1 modification of VHL will have a major impact on its inactivity as a ubiquitin ligase. However, the potential of SUMOylation resulting in the inhibition of VHL ubiquitylation will provide a mechanism which distinguishes between substrate ubiquitylation and self-ubiquitylation. The identification of PIASy mediated VHL SUMOylation and instead of ubiquitylation now opens the possibility that PIASy is a component of a finely balanced regulatory process in response to hypoxic stress. To our knowledge factors that transmit conflicting signals to VHL is a rarity. It is therefore tempting to speculate that PIASy is a key player contributing to transmission and integration of signals to inactivate VHL-mediated inhibitory functions on both HIFα and other molecules. The duration of protein SUMOylation is expected to reflect the time frame during which the ubiquitylation of VHL occurs. A temporal increase in VHL SUMOylation occurs in response to hypoxia and is inversely correlated with elevated levels of HIF1α. The fact that attenuated VHL SUMOylation by inhibition of PIASy is likely to explain why VHL is still capable of mediating the efficient destruction of HIFα during hypoxia.

In addition, because there are two translational initiation codons for VHL mRNA [47], [48]. The VHL mRNA produces two native proteins: full length VHL and the shorter VHL_19_ with the first 53 residues deleted [47], [48]. Studies have shown that both proteins are functionally active and the majority of the disease-associated mutations are located downstream of the second translational initiation site [48]. Mutations in the first 53 codons of the VHL gene have no effect on the structure of the shorter VHL protein. However, our studies showed that only full length VHL but not VHL_19_ significantly interacts with PIASy, suggesting that there are distinct biological functions between these two isoforms of VHL and that PIASy association is most likely a critical one. Interestingly, recent studies have shown that concurrent mutations in the first 53 codons do contribute to VHL associated diseases [49], which is significant for the specific interaction of PIASy with full length VHL in an adaptive response to hypoxia stress.

## Materials and Methods

### DNA constructs, antibodies and reagents

Plasmids encoding human PIAS protein family (1, 3, x, and y) with FLAG tag were gifts from Stefan Müller (Max Planck Institute of Biochemistry, Germany), and Ke Shuai (University of California Los Angeles, USA). PIASyΔ1-157, PIASyΔ183-415, PIASy1-233, PIASy1-415, PIASy1-461, and PIASy1-492 with FLAG tag were generated by ligating *Bam*HI/*Eco*RV PCR fragments into the plasmid of pcDNA-FLAG. PIASy 1-492 with myc tag were generated by ligating *Bam*HI/*Eco*RV PCR fragments into the pA3M vector. GST-PIASy1-492 was generated by ligating *Bam*HI/*Eco*RI PCR fragments into the pGEX-2TK vector. Plasmids HA-VHL and FLAG-VHL were provided individually by Kaelin WG Jr. (Harvard Medical School) and Joan W Conaway (Stowers Institute for Medical Research, USA). The truncated mutants of HA-VHL Δ1-53, HA-VHL 54-190, HA-VHL 54-151, HA-VHL 54-120 and HA-Ubc9 were constructed by ligating *Bam*HI/*Eco*RI PCR fragments into the plasmid of pcDNA3HA. Constructs of HA-VHL1-120, HA-VHL1-170, and HA-VHL1-190 (which derived from wild type HA-VHL by introducing TGA stop codon as indicated position), FLAG-VHL (K171R, K196R, or K159R), PIASy^C342/347A^-myc and VHL-SUMO1^ΔC4^ (Mut1 and Mut2) were individually generated by PCR site-directed mutagenesis. VHL-Ub^ΔGG^ and VHL-SUMO1^ΔC4^ were generated by in-frame ligation of *Bam*HI/*Xba*I PCR fragments to the downstream of FLAG-VHL (K171R). All constructs were confirmed by direct DNA sequencing. Constructs of pCEP4/HIF1α and wild type HIF1α target reporter pGL2-wHRE, pGL2-mHRE, pGL3-1.5VEGFp, and p53-myc were previously described [50], [51]. The VHL antibodies were from Cell signaling technology Inc. SUMO1 (Y299) antibodies were from Abcam. HIF1α antibodies were from BD transduction laboratory. PIASy (I-19) was purchased from Santa Cruz Biotech. Inc. Other antibodies used were anti-myc (9E10), anti-FLAG (M2), anti-HA (12CA5), and β-Actin (Cell signaling technology). The deSUMOylation inhibitor *N*-Ethylmaleimide (NEM) was purchased from Sigma. Proteasome inhibitor MG132 was purchased from Biomol International.

### Cell culture *and* transfection

Human renal carcinoma *VHL*-null cell lines 786-O and RCC4 (Kindly provided by Dr Volker H. Haase from University of Pennsylvania Medical School of Medicine, Philadelphia, PA), osteosarcoma *p53*-null cell line Saos-2 (Kindly provided by Dr Jon Aster from Brigham and Women's Hospital, Boston, MA), and embryonic kidney (HEK) 293 cells were maintained in Dulbecco's modified Eagle's medium (DMEM) supplemented with 5% fetal bovine serum (FBS, Hyclone), 4 µM L-glutamine, penicillin, and streptomycin [51]. All cells were incubated at 37°C in a humidified environmental incubator supplemented with 5% CO_2_. Ten million cells with 400 µl medium were transfected by electroporation with a Bio-Rad Gene Pulser in 0.4 cm-gap cuvettes at 210 Volts and 975 microfarads or calcium phosphate transfection [51].

### Hypoxic treatment

Cells were cultured at 37°C and 5% CO_2_ in air to reach less than 50% confluency before being transferred to an exposure chamber, which was flushed with 1% O_2_ balanced with 5% CO_2_ and 95% N_2_. The chamber was then sealed airtight and kept at 37°C for the duration of hypoxia treatment [50]. For chemical hypoxia, cells were exposed to 100 µM Cobalt Chloride (Sigma) [50].

### Immunoprecipitation and Immunoblotting

Immunoprecipitation (IP) and immunoblotting (IB) assays were performed as described previously [51]. Briefly, cells were harvested and washed once with ice-cold phosphate-buffered saline (PBS) and lysed in 1 ml cold radioimmunoprecipitation assay (RIPA) buffer (50 mM Tris [pH 7.6], 150 mM NaCl, 2 mM EDTA, 1% Nonidet P-40, 1 mM PMSF, leupeptin [1 µg ml^−1^], aprotinin [1 µg ml^−1^], and pepstatin [1 µg ml^−1^] with or without 10 mM *N*-ethylmaleimide (NEM)]) on ice and homogenized. The supernatant of lysates were pre-cleared with protein A/G Sepharose beads (Amersham Biosciences, Piscataway, N.J.); and then incubated with primary antibody 2 h at 4°C with constant rotation and then with protein A/G Sepharose beads for 1 h. Beads were washed 5 times with TBS buffer, resuspend in 50 µl of 1× SDS Laemmli buffer and heated 95°C for 5 minutes. The sample was subjected to SDS-PAGE and transferred to a membrane that was probed with specific antibody. For denatured IP, cells were boiled 10 min at 1% SDS-containing Tris-buffered saline, followed by sonication and 18-fold dilution with TBS containing 1% Triton X-100. The cell lysates were immunoprecipitated with SUMO1 (Y299) antibody [21].

### Protein expression, and *in vitro* pull-down assay

GST and GST-PIASy were expressed in BL21 (DE3) *E.coli* cells and purified as previously described[51]. Briefly, overnight starter cultures (50 ml) of *Escherichia coli* BL21 (DE3) transformed with pGEX-2T-PIASy mutants were incubated into 500 ml of culture medium and grown at 30°C to an optical density of about 0.6 at 600 nm. After isopropylthiogalactopyranoside (IPTG) induction (0.5 mM, 2 hrs at 30°C), for GST-fused protein, bacteria were collected and sonicated in lysis buffer containing 20 mM Tris-HCl pH 8.0, 100 mM NaCl, 0.5% NP40, 1 mM EDTA, 1 M DTT, 5% Sarkosyl and the protease inhibitor cocktail. For pull-down assay, ^35^S-methionine-labeled *in vitro*-translated proteins were incubated with the relevant GST-fusion proteins loaded on glutathione sepharose bead for 4 hrs at 4°C in GST binding buffer (50 mM Tris-HCl pH 7.5, 100 mM NaCl, 10 µm ZnCl_2_, 10% glycerol, freshly supplemented with 0.1 mM dithiothreitol (DTT) and protease inhibitor). After washing, bound proteins were eluted with SDS sample buffer and analyzed by gel electrophoresis followed by direct antuoradiography and scan by Phospho Imager (Amersham Biosciences Inc., Piscataway, NJ).

### *In vitro* SUMOylation assay

*In vitro* SUMOylation kit was purchased from BostonBiochem Incorp. (Cambridge, MA). The reaction was carried out at 37°C for 1 hr with the mixture including E1 (100 nM), E2 (5 µM), SUMO1 (50 µM), ATP (1 mM), and ^35^S-methionine-labeled *in vitro*-translated wild type FLAG-VHL or its mutants (2 µl) in total 20 µl volume.

### Oligomerization assay

*In vitro* translated, radiolabeled proteins (3 µl) were resuspended in NETN buffer (20 mM Tris-HCl pH 8.0, 100 mM NaCl, 0.5% NP40, 1 mM EDTA, and the protease inhibitor cocktail), and then incubated on ice for 15 min with or without 0.1% glutaraldehyde[52]. Proteins were then resolved by SDS-PAGE and analyzed by autoradiography.

### Luciferase reporter assay

Luciferase reporter plasmids pGL2-wHRE and pGL2-mHRE (pGL2, containing 3 tandem repeats of HIF-binding sites or its mutants) were gifts from Gregg Semenza (Johns Hopkins University). The luciferase reporter assays were performed as described previously[51]. After transfection for 48 h, cells were lysed in 200 µl of reporter lysis buffer (Promega, Inc., Madison, WI). Luciferase activities and β-galactosidase were individually measured using luciferase assay reagent (Promega, Inc., Madison, WI) and the OpticompI Luminometer (MGM Instruments, Inc. Hamden, CT) according to the suppliers' instructions. Luciferase activities were normalized with β-galactosidase activities. Relative luciferase activity (RLU) was expressed as fold activation relative to the reporter construct alone. Assays were performed in triplicate.

### RNA interference

For RNA interference with luciferase reporter assays, the PIASy shRNA sequence (5′-GTACTTAAACGGACTGGGA-3′), Ubc9 shRNA sequence (5′-GGGAAGGAGGCTTGTTTAAAC-3′), and the control sequence (5′-TGCGTTGCTAGTACCAAC-3′, non-targeting sequence), were cloned into RNAi-Ready pSIREN (Clontech). For the Lentivirus-mediated stable knockdown of PIASy, the PIASy shRNA sequence was inserted into pGIPz vector according to the manufacturer's instructions (Clonetech), 293 and 786-O cells were individually transduction by Lentivirus packaged from Core T which cotransfected with Rev, VSVG and gp expressing plasmids, and selection by 1 µg/ml Puromycin. pGIPz vector with luciferase target sequence (shLuc) was used as control, and the RNA interfering efficiency was assessed by western blot analysis.

### Endothelial tube formation assay

Human umbilical vascular endothelial cells (HUVEC) were purchased (Cambrex) and maintained in EBM-2 medium supplemented with EGM-2. Tube formation assay on extracellular BD matrigel was performed according to the manufacturer's protocol with minor modifications. Briefly, 2×10^4^ HUVEC resuspended with 500 µl of EBM-2 medium were seeded on Matrixgel solidified in 48-wel tissue culture plate. Conditioned medium (1 ml) from the supernatant of each 786-O stable cells was added, respectively. Cells were incubated in a CO_2_ incubator for 24 hrs at 37°C and then examined for tube formation with a light microscope.

### Flow cytometry, cell cycle assay

Cells were transfected with an empty vector, wild type FLAG-VHL or its mutants in 100-mm dishes. After incubation, cells were harvested and washed twice with PBS. They were fixed by treatment with 1 ml of 70% ethanol, gently vortexed and kept at 4°C until used. Fixed cells were washed once with PBS and resuspend in a propidium iodide solution (10 µg ml^−1^) containing RNase A (250 µg ml^−1^). Propidium iodide-stained cells were then analyzed for their DNA contents by using a FACSCalibur cytometer (Becton Dickinson, San Jose, CA) and FlowJo software (Tree Star, Ashland, OR).

### Characterization of cell growth in vitro

Cells transfected with wild type VHL and its mutants were selected by G418 (1 mg ml^−1^) and 2×10^5^ cells were plated into 6-well plates. Cell numbers were counted with a hemocytometer (Beckman Coulter) at each time point for 10 days. Cell counts were performed in triplicate.

### Cell migration assay

Cell mobility was assessed using a scratch wound assay. Stably transfected cells were cultured in 6-well plates until confluent. The cell layers were carefully wounded using sterile tips and washed twice with fresh medium. After incubation for 12, 24 and 36 hrs, the cells were photographed under a phase-contrast microscope. The experiments were performed in triplicate.

### 3D multicellular tumor spheroid assay

For multicellular spheroids were prepared as described previously [29]. Briefly, 48-well tissue culture plates were coated with 50 µl of prewarmed 0.5% soft agrose in serum-free medium. A total of 10^4^ transfected cells with G418 (1 mg ml^−1^) selection were resuspended in 0.5 ml of DMEM supplemented with 10% fetal calf serum and added to the agrose-coated wells. The cells were grown in a humidified 5% CO2 atmosphere at 37°C for 3 days. Digital images were taken at ×40 magnification.

## Supporting Information

Figure S1 The homologous sequence with potential SUMO modified sites in VHL from different species. AD, acidic domain; m, mouse; r, rabbit; d, dog, M, monkey; h, homo sapiens.
(1.46 MB TIF)

Figure S2 (A) Crosslinking assays in vitro. VHL, VHL-SUMO1dC4 and VHL-UbdGG proteins were individually in vitro translated, radiolabelled, and treated with different concentration glutaraldehyde (GA) and analyzed by SDS-PAGE and autoradiography. In vitro translated, radiolabelled p53-myc protein was used as positive control. (B) The stability analysis of dimerized VHL-SUMO1dC4 in vitro. The in vitro translated proteins were individually subjected to treatment as indicated in the figure, and analyzed by SDS-PAGE and autoradiography.
(3.44 MB TIF)

Figure S3 Beta domain of VHL binds to PIASy in vitro. The purified GST-PIASy proteins (4 µg each) were incubated individually with 12 µl in vitro translated 35S-labeled wild type HA-VHL and its mutants at 4°C for 4 h. After extensive washing, the samples were resolved by SDS-PAGE and detected by a Phosphor Imager. The input of VHL and luciferase (luc) as negative control are shown.
(1.81 MB TIF)

Figure S4 (A) Loss of PIASy reduces the stability of VHL-SUMO1ΔC4 in hypoxia. 786-O stable cells with PIASy knockdown or luciferase control were individually transfected with VHL-SUMO1ΔC4. At 24 hr posttransfection, cells were equally divided and treated with 1% O2 for 0, 6, 12 or 24 hours. The levels of VHL-SUMO1ΔC4 were detected by immunoblotting with anti-FLAG. (B) Loss of PIASy but not Ubc9 attenuates the transcriptional activity of HIF-responsive reporter. HEK293 cells were transiently transfected with pSIREN expressing small hairpin against PIASy or Ubc9 in the presence of wild type (wHRE) or mutation (mHRE) HIF-responsive element-luciferase reporter. Empty vector was used as control. Data are presented as means±SD of three independent experiments.
(2.57 MB TIF)

Figure S5 (A) Loss of PIASy enhances the effect of VHL positive but not negative cells on cell cycle under serum starve stress. Cells were plated and incubated for 18 hrs in either 1% oxygen or 0.1% serum, and directly analyzed by flow cytometry after staining with propidium iodide. The relative ratios of G0 phase in each group were presented by compared to luciferase knockdown (shLuc) control under normal condition. a, HeLa; b, 293; c, 786-O; d, RCC4. (B) Loss of PIASy suppresses the cell mobility in normoxia. The cell mobility of HeLa cells with PIASy stable knockdown or luciferase control in normoxia were determined by wound migration assay as described previously.
(2.34 MB TIF)
