# Supplementary figures and images for: Hypoxia Inactivates the VHL Tumor Suppressor through PIASy-Mediated SUMO Modification
Source: PLoS One. 2010 Mar 16;5(3):e9720. doi: 10.1371/journal.pone.0009720 (PMC2838797; doi:10.1371/journal.pone.0009720)

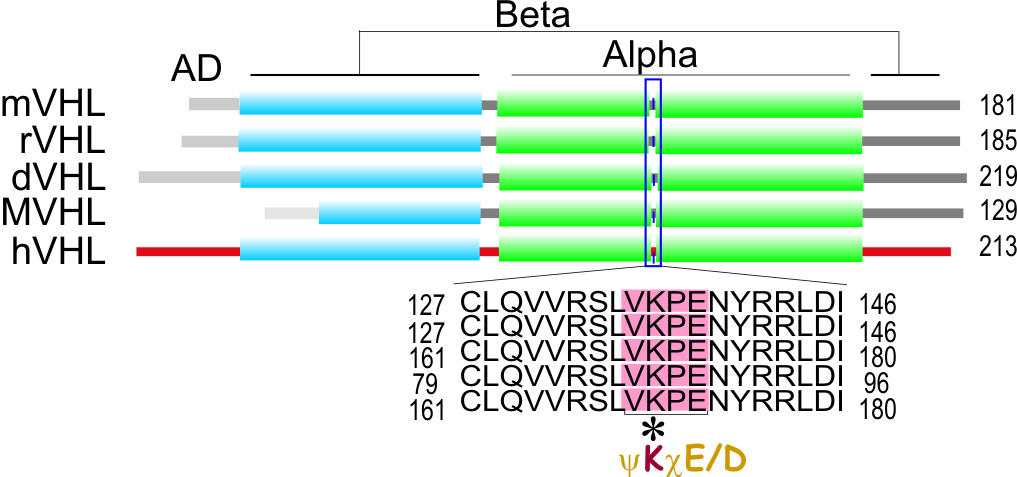

Supplement: Figure S1 — The homologous sequence with potential SUMO modified sites in VHL from different species. AD, acidic domain; m, mouse; r, rabbit; d, dog, M, monkey; h, homo sapiens. (1.46 MB TIF) [file pone.0009720.s001.tif]

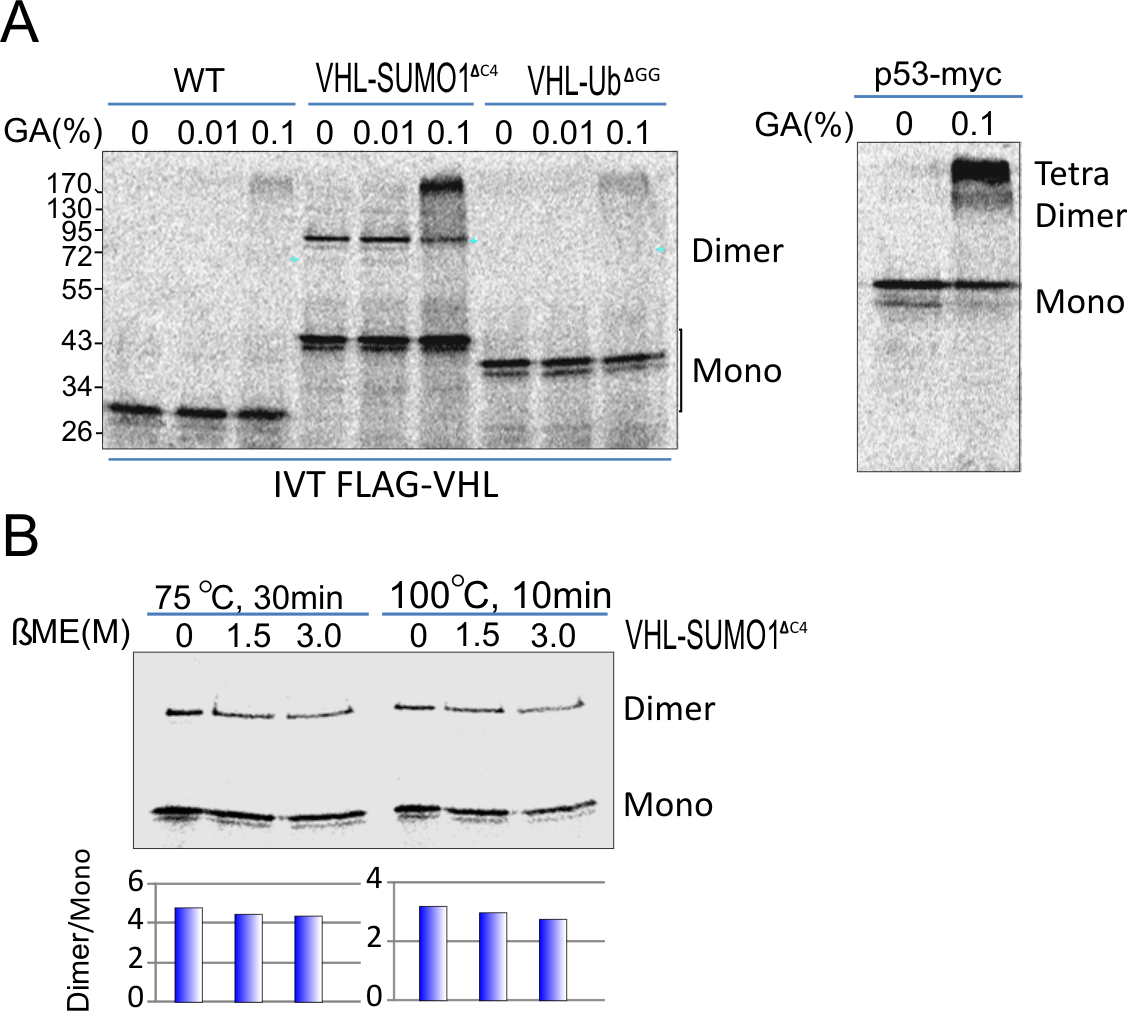

Supplement: Figure S2 — (A) Crosslinking assays in vitro. VHL, VHL-SUMO1dC4 and VHL-UbdGG proteins were individually in vitro translated, radiolabelled, and treated with different concentration glutaraldehyde (GA) and analyzed by SDS-PAGE and autoradiography. In vitro translated, radiolabelled p53-myc protein was used as positive control. (B) The stability analysis of dimerized VHL-SUMO1dC4 in vitro. The in vitro translated proteins were individually subjected to treatment as indicated in the figure, and analyzed by SDS-PAGE and autoradiography. (3.44 MB TIF) [file pone.0009720.s002.tif]

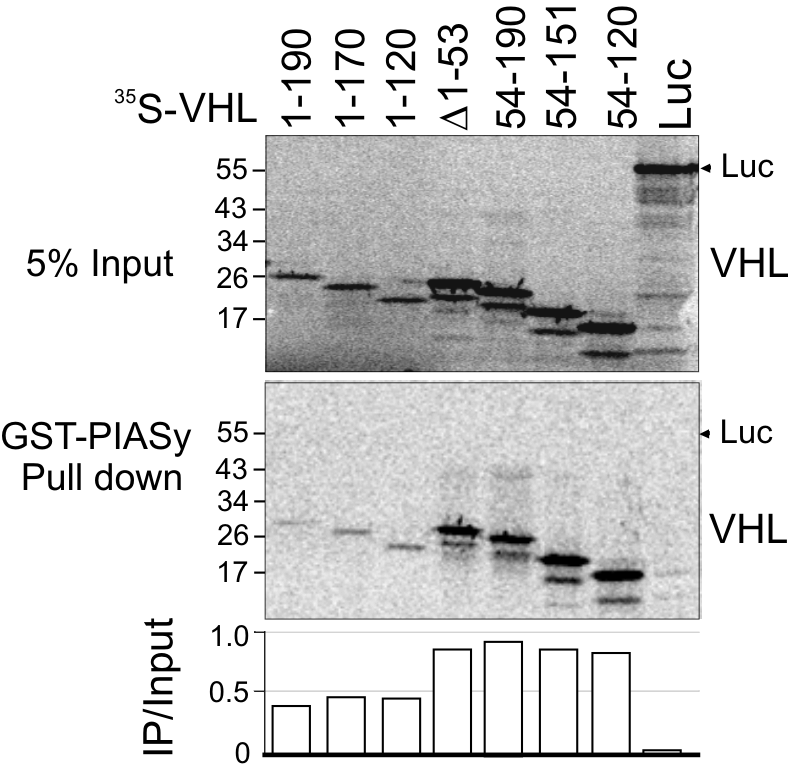

Supplement: Figure S3 — Beta domain of VHL binds to PIASy in vitro. The purified GST-PIASy proteins (4 µg each) were incubated individually with 12 µl in vitro translated 35S-labeled wild type HA-VHL and its mutants at 4°C for 4 h. After extensive washing, the samples were resolved by SDS-PAGE and detected by a Phosphor Imager. The input of VHL and luciferase (luc) as negative control are shown. (1.81 MB TIF) [file pone.0009720.s003.tif]

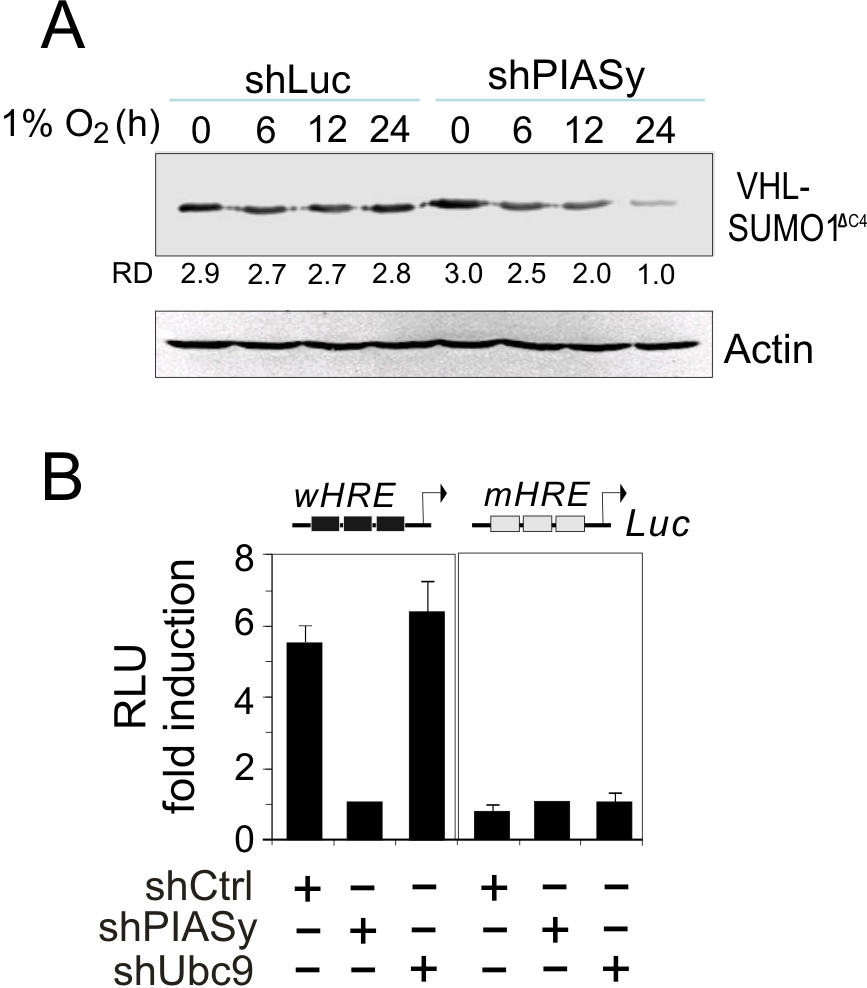

Supplement: Figure S4 — (A) Loss of PIASy reduces the stability of VHL-SUMO1ΔC4 in hypoxia. 786-O stable cells with PIASy knockdown or luciferase control were individually transfected with VHL-SUMO1ΔC4. At 24 hr posttransfection, cells were equally divided and treated with 1% O2 for 0, 6, 12 or 24 hours. The levels of VHL-SUMO1ΔC4 were detected by immunoblotting with anti-FLAG. (B) Loss of PIASy but not Ubc9 attenuates the transcriptional activity of HIF-responsive reporter. HEK293 cells were transiently transfected with pSIREN expressing small hairpin against PIASy or Ubc9 in the presence of wild type (wHRE) or mutation (mHRE) HIF-responsive element-luciferase reporter. Empty vector was used as control. Data are presented as means±SD of three independent experiments. (2.57 MB TIF) [file pone.0009720.s004.tif]

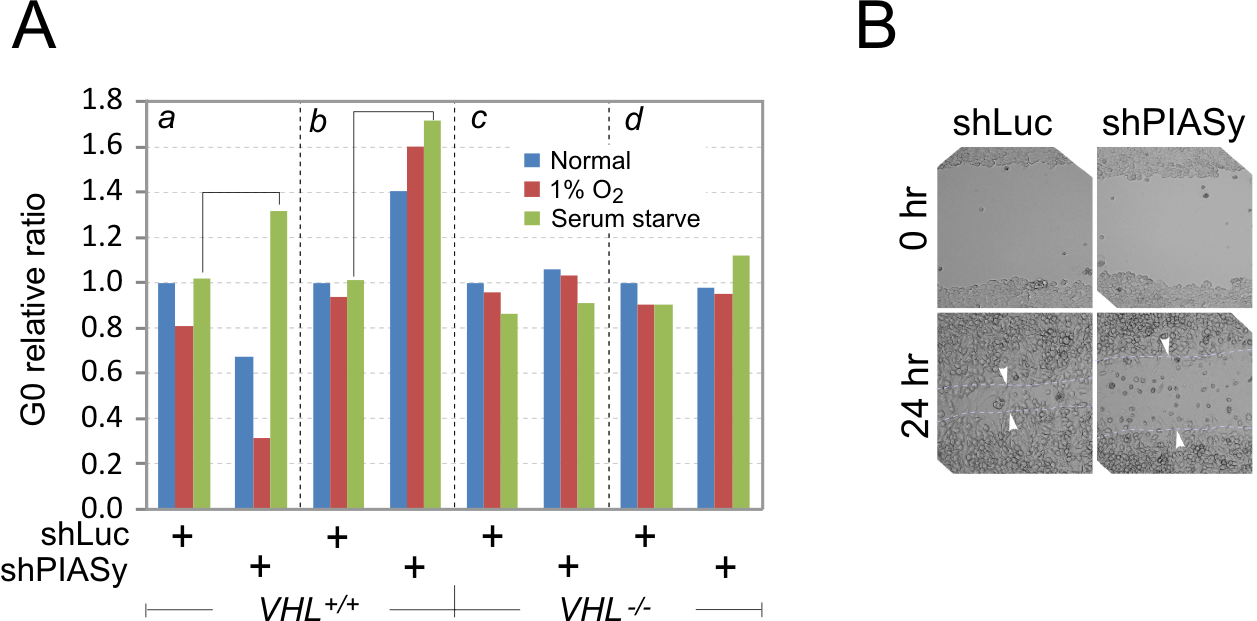

Supplement: Figure S5 — (A) Loss of PIASy enhances the effect of VHL positive but not negative cells on cell cycle under serum starve stress. Cells were plated and incubated for 18 hrs in either 1% oxygen or 0.1% serum, and directly analyzed by flow cytometry after staining with propidium iodide. The relative ratios of G0 phase in each group were presented by compared to luciferase knockdown (shLuc) control under normal condition. a, HeLa; b, 293; c, 786-O; d, RCC4. (B) Loss of PIASy suppresses the cell mobility in normoxia. The cell mobility of HeLa cells with PIASy stable knockdown or luciferase control in normoxia were determined by wound migration assay as described previously. (2.34 MB TIF) [file pone.0009720.s005.tif]
